# Improving Serious Games to Tackle Childhood Obesity

**DOI:** 10.3389/fpsyg.2021.657289

**Published:** 2021-05-06

**Authors:** Maroua Belghali, Yauhen Statsenko, Abdulsalam Al-Za’abi

**Affiliations:** ^1^Department of Health and Physical Education, College of Education, United Arab Emirates University, Al-Ain, United Arab Emirates; ^2^College of Medicine and Health Sciences, United Arab Emirates University, Al-Ain, United Arab Emirates

**Keywords:** childhood obesity, executive functions, serious games, nutrition education, physical exercise

## Abstract

Childhood obesity has become a global public health issue. Today, there are opportunities to promote health through technological devices such as serious games. Despite the major advancement of this field of research, the use of serious games as a validated intervention in clinical practice requires further clarifications on some methodological aspects. In this perspective article, we report the pros and cons of existing serious games. Besides, we attempt to propose a new methodology of design of a serious game that could help to cope with childhood obesity. The proposed idea consists of a serious game in virtual reality based on enjoyment, movement, education, and executive functioning (EF) training. Longitudinal studies and solid research protocol would certainly ensure consistency and aid interpretation.

## Introduction

The prevalence of obesity among children is freighting, and is constantly raising in both developed (where 24% of boys and 23% of girls are overweight or obese; Ng et al., 2014) and developing countries (where 13% of both boys and girls are overweight or obese; Ng et al., 2014). For example, in the United Arab Emirates, more than 41.2% of children were overweight in 2016, and 19% of these were obese (AlBlooshi et al., 2016). The causes of obesity are multifactorial (Ang et al., 2013); including unhealthy eating patterns and a lack of physical activity (PA; Hill et al., 2012), both of which may relate to an increase in the time spent watching media (e.g., TV) and playing videogames (Lamboglia et al., 2013). More recently, advances in neuroscience and neuropsychology showed that neurocognitive factors may also play a causal-role in obesity (Deary et al., 2002; Gottfredson and Deary, 2004), especially executive functioning (EF; Hall et al., 2008). EF is a central aspect of self-regulation that is necessary to manage behavior, caloric intake, and PA in an obesogenic environment (Russell and Russell, 2020). Thus, if EF is deficient, it may be difficult to employ efficient behavior (e.g., choosing healthy foods, etc.) that aid in maintaining energy balance, leading to an increase in body weight over time (Naets et al., 2018).

Currently, the accepted treatment regime for the management of childhood obesity is a multidisciplinary intervention targeting lifestyle behaviors at both the individual and familial levels (Naets et al., 2018; Del Río et al., 2019), to (1) reduce calories, while improving diet, (2) reduce sedentary behavior, and (3) increase physical exercise. Sedentary behavior was defined as, “*any waking behavior characterized by energy expenditure ≤1.5 metabolic equivalents (METs), while in a sitting or reclining posture*” (Sedentary Behaviour Research Network, 2012). Notably, screen time and sitting time were used in the majority of studies as two main parameters to both quantify and reduce the time devoted to sedentary behaviors (Minges et al., 2015). With regards to physical exercise, most studies relied on aerobic and anaerobic exercises, which may increase the daily energy expenditure (i.e., ≥3 METs) and provide weight loss (Keadle et al., 2017).

While the multidisciplinary approach had a clinically significant impact on body weight, physical fitness, and psychosocial wellbeing, its long-term success was modest and not sustained (Naets et al., 2018). This signifies the urgent need to implement novel intervention strategies for the long-term treatment of childhood obesity. In the light of these perspectives, health researchers suggest the use of technological devices (Trost et al., 2014; Staiano et al., 2018) including serious games, as these catch children’s motivation to maintain the adherence to intervention (Dias et al., 2018; Baranowski et al., 2019). Serious games were defined as “games designed to persuade players to modify their health-related attitudes or behaviors through playing and entertainment” (Fonseca et al., 2017).

In the context of obesity, there are four types of serious games: The first type aimed to decrease energy intake by improving knowledge concerning healthy nutrition and changing children’s attitudes about food (i.e., serious games based on nutrition education and/or dietary change). The second type aimed to reinforce the weight control process and apply nutrition knowledge in daily life (serious games based on EF training). The third type aimed to increase the energy expenditure (i.e., serious games based on physical activity: exergames). The last type aimed to provide knowledge, enhance motivation, and encourage behavior change related to healthy eating, PA, and stress coping (i.e., multidisciplinary serious games). Current studies showed that playing serious games might contribute to tackling childhood obesity (Mack et al., 2020; Ruggiero et al., 2020). Despite the major importance of this finding, the use of serious games as a validated intervention in clinical practice requires further clarifications on some methodological aspects (Lu et al., 2013; Dias et al., 2018). In this perspective article, we reported the pros and cons of serious games dedicated to treating childhood obesity. Besides, we attempt to propose a new methodology of design of a serious game that could help to cope with obesity and enhance its use in clinical practice.

## Serious Game Based on Nutrition Education and/or Dietary Change: Pros and Cons in Tackling Childhood Obesity

Serious games based on nutrition knowledge and/or dietary change were designed primarily to reduce energy intake by (i) improving knowledge concerning healthy nutrition, and (ii) changing children’s attitudes about food. Lu et al. (2013) and Dias et al. (2018) conducted two systematic reviews and identified six studies published between 2003 and 2018. Accordingly, children with obesity manifested great interest in serious games compared to standard interventions (e.g., printed pamphlets addressing diet). Specifically, five published trials effectively improved knowledge and eating habits including fruit and vegetable consumption, whereas one did not, mainly because some participants did not complete the intervention as expected. Another possible explanation is that the participants of this study were allowed to play without researcher monitoring. More recently, Del Río et al. (2019) carried out a long-term longitudinal study of 3 years on 46 children with obesity and found significant improvements between the experimental and control groups in terms of their knowledge of healthy nutrition and their adherence to the mediterranean diet, after playing a gamified educational program for healthy habits, based on active video games and motor games. While most studies demonstrated some positive effects on nutrition knowledge and eating habits, the effect of this type of serious games on body composition was less frequently assessed and the results were inconsistent (see Dias et al., 2018 for a review).

Despite the relevance of these findings, there is a need to standardize how serious games were assessed. Another critical point is that the “source” of eating habits in the majority of studies is not the children but rather the parents (Thompson et al., 2010). Hence, children’s eating habits may face their parents’ absence of knowledge about healthy eating behavior (Thompson et al., 2010). This fact creates a huge obstacle to the construction of a healthy lifestyle that will help to lose weight in a long lasting-way (Thompson et al., 2010). One solution to this matter lays in (i) making the children aware of the need to change their attitudes toward food choice (Druzhinenko et al., 2014; Sirico et al., 2018), and (ii) the willingness to change the actual behavior (i.e., “conscious intention”; Thompson et al., 2010). Taking together, we suggest that integrating both awareness and real behavioral techniques into serious games may help to achieve long-term real-world effectiveness.

## Serious Games Based on Executive Functioning Training

Executive functioning regroups higher cognitive processes allowing flexible behavior to environmental circumstances in essentially all facets of daily living (Miyake and Friedman, 2012). EF is regulated by the prefrontal cortex and involves three main components: (1) inhibition (i.e., deliberate overriding of dominant or prepotent responses; Miyake and Friedman, 2012), (2) working memory (i.e., the ability to both hold and monitor different information modalities; Miyake and Friedman, 2012); and (3) switching (i.e., switching flexibly between tasks or mental sets; Miyake and Friedman, 2012). The available evidence suggest a causal role of EF in the development of childhood obesity. For instance, the longitudinal study by Moffitt et al. (2011) showed that problems in EF measured in children age 3–11, were associated with weight-related problems, at a 32-year follow-up. This is mainly because problems in EF may hinder the development of skills necessary to resist food temptation, leading to binge eating episodes and, obesity, consequently (Gettens and Gorin, 2017). Other studies reported that EF was a stronger predictor of healthy dietary choice (Hall et al., 2008), and was the only significant predictor of high fat intake, as well as fruit and vegetable consumption at 1 year when included in a model with conscientiousness, and many other personality characteristics (Hall and Fong, 2013). EF also may plays an important role in the treatment of obesity (Naets et al., 2018). Some studies showed that children with EF deficiency experienced more difficulties in weight loss and weight loss maintenance, both in the short and long terms, and prematurely drop out of intervention more often (Hjördis and Gunnar, 1989; Kulendran et al., 2014; Weygandt et al., 2015; Naets et al., 2018). It seems likely that, as long as children do not strengthen their EF, their self-control strategies remain of limited capacity (Verbeken et al., 2013; Houben et al., 2018).

Taking into account all the above, a burgeoning field of research suggests that training EF may be a promising strategy to aid in obesity treatment (Jones et al., 2018). Despite the relevance of this finding, only one research group (Verbeken et al., 2013) took the initiative to develop a serious game (i.e., called “Braingame Brian”) based on EF-training for children with obesity. The “Braingame Brian” combined inhibition and working memory. Results from this study showed that training sessions were well tolerated for the children with obesity. More interestingly results showed improvement not only in the children’s EF skills but also, compared to the control group, the children who completed the EF-training appeared to be more capable of maintaining their weight-loss and healthy lifestyle behavior, up to 6-weeks after finishing a 10-month inpatient treatment program. This finding suggests that the serious game was sufficient to alter the underlying EF mechanisms that facilitate weight-loss maintenance behaviors. Beyond this explanation, Gettens and Gorin (2017) proposed an elegant conceptual model of the relationships between EF, initial weight loss, and weight loss maintenance, mapping specific EF onto strategies known to be associated with the weight control process.

## Exergaming: Pros and Cons in Tackling Childhood Obesity

Exergaming is an emerging technology that allows the players to interact physically with onscreen avatars through a variety of gross motor movements such as jumping, kicking, punching, and ducking (Zeng and Gao, 2016). Today, there are many types of exergames in the market (i.e., commercial exergames), including, but not limited to the Nintendo Wii Fit, Xbox Kinect, and the Dance Dance Revolution. It is worth mentioning that commercial exergames requiring the use of the Kinect Sensor (i.e., a device capable of recognizing the human body and its environment) are not available anymore (e.g., Kinect, Nintendo Wii; Bonnechère et al., 2018). This may return children to passive video games and lead to bad effects on children’s health such as obesity. With this regard, Bonnechère et al. (2018) highlighted the urgent need to create a large consortium of clinicians, researchers, hardware developers, and industrials to develop alternative solutions. In this perspective, some authors took the initiative to develop their own exergames based on specific theories including the Social Cognitive Theory (Staiano et al., 2012); validated them, and later applied them to children with obesity (e.g., Espinosa-Curiel et al., 2020).

Systematic reviews and meta-analyses comparing the effects of playing exergames to engaging in another form of sedentary screen time on obesity-related outcomes reported inconsistencies in the literature (Lamboglia et al., 2013; Zeng and Gao, 2016). Some laboratory-based studies and field-based studies reported short-term benefits on the (i) physical (e.g., improvements in the levels of PA and functional fitness; Gao and Chen, 2014; Vernadakis et al., 2015), (ii) adiposity (e.g., decreases in the body mass index (BMI), percentage of body fat and fat mass; Lamboglia et al., 2013; Gao and Chen, 2014; LeBlanc et al., 2014; Staiano et al., 2018), (iii) physiological (e.g., improvements in the aerobic fitness, and maximal oxygen consumption; Maddison et al., 2011; Staiano et al., 2018), and (iv) psychological outcomes (e.g., improvements in the self-efficacy and social competence; Zeng and Gao, 2016; Andrade et al., 2019), while some reported null findings (see Guy et al., 2011 and LeBlanc et al., 2013 for systematic reviews), and some presented negative associations between exergames and obesity-related health outcomes (LeBlanc et al., 2014). This inconsistency may be the result of the variety of caloric cost of exergames, measurement methods, and the different research designs adopted in the studies. For instance, the meta-analysis by Gao et al. (2015) showed that children benefit from exergames only when the interventions were longer than 8 weeks. Another possible explanation is that some authors involved exergames as part of a multidisciplinary protocol, while others used them as a sole intervention. Therefore, comparative research is needed to refine the efficiency of exergames.

Recently, the Youth Compendium of Physical Activities reported PA intensities from exergames play based on the specific type of exergames, age, and gender (Butte et al., 2018). Accordingly, exergames can be a tool to stimulate PA and can reach levels of moderate- intensity activity for Nintendo Wii Fit exergames (i.e., ≥3 METs) to vigorous-intensity activity for action running exergames (i.e., ≥8 METs; Butte et al., 2018; Norozi et al., 2020). Moreover, based on a meta-analysis of 35 studies, exergames were greater in intensity than laboratory-based exercise activities such as brisk walking and biking, but lower than laboratory-based running (Gao et al., 2015). Although some exergames can acutely increase moderate-to-vigorous-intensity PA in motivated players, they typically do not elicit activity of a high enough intensity, or for a long enough period to enable children with obesity to meet PA guidelines (Biddiss and Irwin, 2010; LeBlanc et al., 2013). The inconsistency in findings may be that the fitness level of the player was not taken into account while playing (Zeng and Gao, 2016). Some exergames required movements, which were not always possible for children with obesity (Hwang et al., 2019). One recent study confirmed this view and showed that children with obesity spent more time at light-intensity but less time at the vigorous-intensity with fewer movements especially, while playing a lower limb–controlled exergames, resulting in sharp declines in exergaming after a few weeks (Hwang et al., 2019). Hence, we can question the advisability of focusing only on the total energy expenditure in the design of exergames without predefining the capacities and limits of each player. From our point of view, applying the different principles of training (i.e., overload, specificity, reversibility, and variance), and the fitness level of the player in the game design may help to meet the recommended level of moderate to vigorous PA among children with obesity.

It is worth mentioning that only a few studies examined the possible effects of exergames on snacking behavior during activity. The study by Mellecker et al. (2010) showed that children increased their energy intake through snack or soft drink consumption regardless of whether they are playing seated video games, or playing exergames in 9–13-year-old children. Specifically, snacking energy intake while playing (seated video games, or exergames) was 166% more than the calories required during resting conditions. This result may suggest that the energy expenditure promoted by exergames could be offset by snaking behavior (Chaput, 2011). More recently, the study by Allsop et al. (2016) contradicted the previous findings and found that the energy intake was significantly greater during seated video gaming than during exergaming in 8–11-year-old boys. This inconsistency may be the result of the variety of the type of activity used in exergaming, and the different research designs adopted in the studies. Moreover, it seems that exergames requiring a higher degree of motor skill and physical intensity would decrease the energy intake while playing. On the other hand, light intensity exergames may provide more opportunities for snaking. More studies are needed to confirm our opinion.

## Multidisciplinary Serious Game

To date, four research groups took the initiative to develop multidisciplinary serious games including different game modules that can be used to promote healthy behaviors in children with obesity. Three of them were recently published (Espinosa-Curiel et al., 2020; Mack et al., 2020; Ruggiero et al., 2020), while one is still in progress (Mâsse et al., 2020). The overall content (i.e., available online) and outcome goals were to provide knowledge, enhance motivation, and encourage behavior change related to healthy eating, PA, and stress coping.

Espinosa-Curiel et al. (2020) demonstrated that 8–10-year-old children improved their nutritional knowledge, increased their intake of healthy food, and reduced their intake of unhealthy food after playing FoodRateMaster. Moreover, the participants’ parents agreed that FoodRateMaster positively influenced their children’s attitudes toward several healthy eating behaviors. Critically, while the FoodRateMaster was designed as an active game to promote PA, the authors did not assess the PA levels of the players. A subsequent study by Mack et al. (2020) assessing the effects of their multidisciplinary game among children aged 8–12 years, indicated that players increased knowledge in the areas of nutrition and stress coping, and were able to apply the dietary energy density principle of nutrition education. The stress coping is an important factor in weight regulation, as stress can be associated with poor dietary habits and low physical activity, especially when an individual faces challenges that surpass his or her coping skills (Mack et al., 2020). No changes at the behavioral level (e.g., media consumption and PA level) occurred because of the study focus (i.e., nutrition education) and design. Ruggiero et al. (2020) assessed the effects of the “MyPlate Picks” game among children aged 7–13 years, indicating that players enhanced their motivation, and increased their nutrition knowledge about healthy eating and PA after 6 weeks of gameplay. Interestingly, findings clarified that the use of a team approach for youth was more acceptable and feasible than individual gameplay.

Critically, the available multidisciplinary serious games focused more on nutrition knowledge and dietary change and much less on PA, which was lightly involved in the game design. The latter also clearly lacked elements of EF training.

## How Could a Serious Game be Improved to Tackling Childhood Obesity?

**Figure 1:**
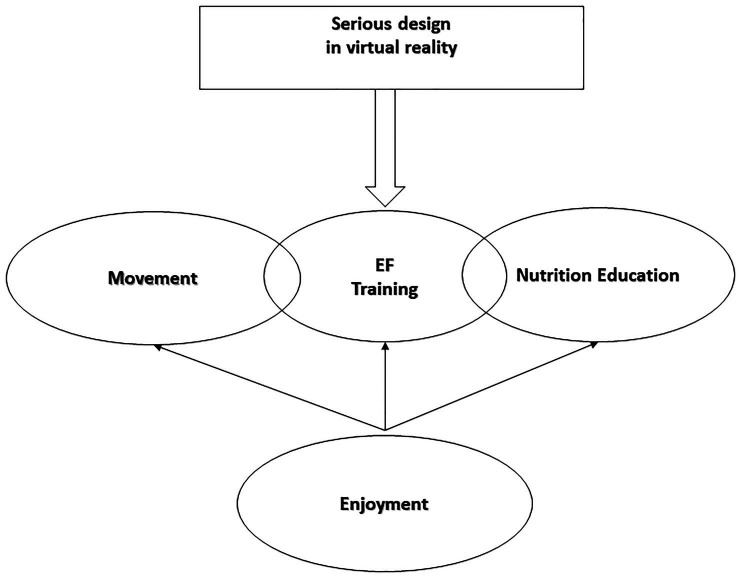
The four-dimensional framework of a serious game in virtual reality: enjoyment, movement, nutrition education, and executive functioning (EF) training.

Serious gaming could be a promising new way to tackle childhood obesity. In this perspective article, we discuss relevant trials on serious games for four main purposes: (1) decrease energy intake, (2) increase energy expenditure, (3) reinforce the weight control process, and (4) encourage behavior change related to healthy eating, PA, and stress coping. Still, none of the published serious games combined all these purposes into the same game design, whereas all of them are essential for successful weight control (Hill et al., 2012). From our point of view, this approach seems to offer the most promising option for tackling childhood obesity in clinical practice. In other words, serious games dedicated to tackle childhood obesity may be improved through a Four-Dimensional Framework: Enjoyment, Movement, Nutrition Education, and EF training (Figure 1).

Applying our new model would be possible by incorporating both healthy and unhealthy eating messages (nutrition education), into the exergames instead of a film game. Moreover, as EF plays an essential role in both learning processes and children’s attitudes about food (Gettens and Gorin, 2017), we suggest that nutrition education may be more efficient when it is perused in the form of an EF training (i.e., please see model of Gettens and Gorin, 2017 for examples).

In respect to the movement dimension, the active gaming mode must contain a properly planned program of PA instead of non-controlled movements to ensure the reliability of the program as well as a child’s safety (FITT principle: frequency, intensity, time, and type). It is important to highlight that the intensity of serious game must range from moderate to vigorous activity. This will help to both prevent and treat childhood obesity (World Health Organization, 2010).

To ensure the enjoyment dimension and reduce stress induced by PA, the use of Virtual Reality appears to offer a good solution due to its fun character (Checa and Bustillo, 2020). Enjoyment is a defining characteristic of intrinsic motivation, which stimulates a release of endorphins and catecholamines (dopamine, serotonin, norepinephrine, and acetylcholine; Mellecker et al., 2013). These neurotransmitters are connected with the brain’s reward system and associated with exercise addiction (Merrer et al., 2009; Mellecker et al., 2013). Moreover, endorphin release may also be a factor in increasing and maintaining PA. Interestingly, Virtual Reality when coupled with PA will help to enhance enjoyment, physiological responses to exercise, and long-term psychological benefits (Please see Mellecker et al., 2013 for a review). In virtual reality, it is also possible to use body sensors and other devices allowing players to be recorded and measured in real time (e.g., psychophysical data: total energy expenditure, cognitive engagement, motor skill complexity… Checa and Bustillo, 2020), thereby offering possibilities for objective measurements that are crucial to test the efficacy of the long-term treatment of childhood obesity such as measures of enjoyment, nutrition-related knowledge, changes in EF, and obesity-related outcomes (i.e., physical, adiposity, physiological, and psychological). It is worth mentioning that the fitness level of the player can be easily extrapolate from non-invasive chronotropic response measurements during low to moderate intensity exercise tests.

Technically, to ensure that each dimension of our model is properly designed, guidelines in the literature must be followed (Carvalho et al., 2015; Michael et al., 2018). In addition, we would argue that it is imperative to take into account factors modulating the player’s performance, among which the most important are age, gender and BMI (Andrade et al., 2019), engagement and intrinsic motivation (Perski et al., 2017), fitness level of the player (Zeng and Gao, 2016), level of immersion in virtual environment (Rose and Chen, 2018), and players’ experience in the virtual reality (Checa and Bustillo, 2020). Serious games intended for children with obesity are often designed to appeal to an expansive age range with little consideration of physical, social, emotional, physiological, and cognitive development. In this perspective article, we suggest that serious games may be targeted at children 7 years and older mainly because children in this age range can understand, perform, and enjoy all aspects of serious gaming. If these methodological recommendations will be taken into consideration, not only the success of the intervention will be more likely, but also the positive long-term effects will increase.

To summarize, creating a serious game in virtual reality based on enjoyment, movement, education, and EF training may have the potential to help treat childhood obesity in clinical practice. Longitudinal studies and standardized protocols would certainly ensure consistency and aid interpretation.

## Data Availability Statement

The original contributions presented in the study are included in the article/supplementary material; further inquiries can be directed to the corresponding author.

## Author Contributions

MB wrote the manuscript. AA-Z and YS revised it. All authors contributed to the article and approved the submitted version.

### Conflict of Interest

The authors declare that the research was conducted in the absence of any commercial or financial relationships that could be construed as a potential conflict of interest.
